# Platelet-Rich Plasma in Sperm Processing for Assisted Reproductive Technology: Molecular Mechanisms, Clinical Applications, and Future Directions

**DOI:** 10.3390/ijms27052177

**Published:** 2026-02-26

**Authors:** Dušica Petrović, Marija Dinić, Dajana Švraka, Veljko Pantović, Emilija Petanovska Kostova, Goran Malenković, Aleksandar Ljubić

**Affiliations:** 1 Pronatal Hospital, Dunavska 2, 11000 Belgrade, Serbia; 2 Department of Transfusiology, University Clinical Center of Serbia, Višegradska 26, 11000 Belgrade, Serbia; klinicka.transfuziologija@gmail.com; 3 Teofanović Hospital, Kralja Bodina 6, 11000 Belgrade, Serbia; info@sgbteofanovic.rs (D.Š.); info@ianubih.bh (A.L.); 4 Nacionalni Centar za Infertilitet I Endokrinologiju Pola, Klinika za Endokrinologiju, Dijabetes I Bolesti Metabolizma, Univerzitetski Klinički Centar Srbije, Doktora Subotića 13, 11000 Beograd, Serbia; klinika.endokrinologija@kcs.ac.rs; 5 Faculty of Medical Sciences, University Goce Delcev Stip, 2000 Štip, North Macedonia; contact@ugd.edu.mk; 6 IVF Centar ReMedika Skopje, 1000 Skopje, North Macedonia; 7 Medicinski Fakultet, Univerzitet u Novom Sadu, Hajduk Veljkova 3, 21000 Novi Sad, Serbia; dekan@mf.uns.ac.rs; 8 International Academy of Sciences and Arts of Bosnia and Herzegovina, Paromlinska 53, 71000 Sarajevo, Bosnia and Herzegovina

**Keywords:** platelet-rich plasma, male infertility, sperm motility, asthenozoospermia, oxidative stress, DNA fragmentation, assisted reproductive technology, ICSI, IVF, growth factors, mitochondrial function

## Abstract

Male infertility contributes to nearly half of infertile couples, with asthenozoospermia and oligoasthenoteratozoospermia as predominant factors. Despite advancements in sperm processing techniques, the outcomes remain limited in severe cases, particularly concerning motility, mitochondrial function, and DNA integrity. Platelet-rich plasma (PRP), an autologous concentrate rich in platelets (>1 × 10^6^/μL) and growth factors, has recently gained attention as an adjunctive therapy in andrology and assisted reproduction. This review systematically evaluated studies published between 2015 and 2025 investigating PRP use in sperm processing, including in vitro experiments, clinical trials, animal models, and mechanistic studies. PRP demonstrated concentration-dependent benefits, with 5% PRP yielding optimal improvements: motility increased by 15–30%, mitochondrial activity increased by up to 80% (*p* = 0.002), and oxidative stress was significantly reduced (*p* < 0.001). PRP’s effects on DNA integrity differ by application method: intratesticular administration improves spermatogenesis, producing sperm with reduced DNA fragmentation (~33% relative reduction after 3 months, *p* < 0.001), while in vitro supplementation provides limited protection against processing-induced damage. Mechanisms involve antioxidant action, mitochondrial protection via AMP-activated protein kinaseNuclear Factor kappa-light-chain-enhancer of activated B cells (AMPK/NF-κB) modulation, membrane stabilization, and the selective preservation of higher-quality spermatozoa. PRP shows consistent biological efficacy and safety but lacks methodological standardization. Fewer than 20% of studies report platelet concentrations, limiting reproducibility. Standardized protocols distinguishing leukocyte-poor from leukocyte-rich preparations and randomized trials focusing on live birth rates are recommended. This review proposes an eight-point characterization checklist for future research.

## 1. Introduction

### 1.1. Male Factor Infertility: Epidemiology and Clinical Impact

Infertility affects approximately 17.5% of couples globally, with male factors contributing to approximately 50% of cases [1]. The World Health Organization estimates that 70 million people worldwide face infertility challenges, representing a significant public health and socioeconomic burden [2]. Male factor infertility encompasses a spectrum of conditions including oligozoospermia (reduced sperm concentration), asthenozoospermia (reduced sperm motility), teratozoospermia (abnormal morphology), and their combinations, with oligoasthenoteratozoospermia (OAT) presenting particularly challenging clinical scenarios.

Among these conditions, impaired sperm motility and DNA integrity represent major causes of reduced fertilization potential, directly affecting the sperm’s ability to traverse the female reproductive tract, penetrate the oocyte, and support embryonic development. Progressive motility, defined as a forward movement of at least 5 μm/second, is the most clinically relevant parameter for both natural conception and assisted reproductive technology success [3]. Equally critical is sperm DNA integrity, as an elevated DNA fragmentation index (DFI) correlates with reduced fertilization rates, impaired embryo quality, increased miscarriage risk, and lower live birth rates across all ART modalities.

### 1.2. Limitations of Current Sperm Processing Techniques

Conventional sperm preparation techniques, while effective at selecting morphologically normal and motile sperm, have inherent limitations that become particularly apparent in cases of severe male factor infertility. Standard laboratory sperm processing methods, including density gradient centrifugation and swim-up techniques, aim to enrich motile spermatozoa through mechanical selection. However, outcomes remain suboptimal in moderate to severe motility impairments and elevated DNA fragmentation, with post-processing recovery being often insufficient for optimal Assisted Reproductive Technology (ART) outcomes.

Repeated centrifugation generates reactive oxygen species (ROS) and mechanical stress, potentially damaging sperm membranes and DNA [4,5]. Furthermore, traditional methods select but do not enhance sperm function; they cannot improve mitochondrial activity, reduce oxidative stress, or prevent DNA damage accumulation. In vitro manipulation and incubation expose sperm to non-physiological conditions, potentially increasing ROS production and cellular damage. When baseline motility is severely compromised (progressive motility < 5%) or DNA fragmentation is elevated (DFI ≥ 30%), even optimal processing yields insufficient functional sperm for conventional insemination or in vitro fertilization.

These limitations have driven the exploration of novel approaches to not merely select, but actively enhance and protect sperm function during processing, with a particular emphasis on preventing the accumulation of additional damage during laboratory handling.

### 1.3. Platelet-Rich Plasma: From Regenerative Medicine to Reproductive Biology

Platelet-rich plasma (PRP) is an autologous blood-derived product containing platelet concentrations significantly elevated above baseline physiological levels, with enrichment factors ranging from 2-fold to 19-fold depending on the preparation technique employed, typically achieving platelet concentrations greater than 1 × 10^6^ platelets/μL for effective clinical PRP (C-PRP) [6,7]. Originally developed for wound healing and tissue regeneration in orthopedic and maxillofacial surgery, PRP has expanded into diverse medical fields [6] including dermatology, dentistry, and cardiovascular medicine based on its demonstrated ability to accelerate tissue repair and reduce inflammation.

The therapeutic potential of PRP derives from alpha-granules within platelets, which upon activation release over 300 bioactive proteins, including multiple growth factors, cytokines, chemokines, and adhesion molecules [8]. These components modulate cellular metabolism, membrane stability, and oxidative stress—processes fundamentally relevant to sperm function. Key growth factors include platelet-derived growth factor (PDGF), which promotes cellular proliferation and migration [9]; vascular endothelial growth factor (VEGF), critical for angiogenesis and cellular metabolism with documented effects on sperm motility [10]; transforming growth factor-beta (TGF-β), which regulates cellular differentiation and immune responses; insulin-like growth factor-1 (IGF-1), which prevents apoptosis through mitochondrial pathway regulation [11]; and epidermal growth factor (EGF) and fibroblast growth factor (FGF), which enhance membrane fluidity and cellular function [12].

In reproductive medicine, PRP has gained attention for treating female infertility, particularly thin endometrium and poor ovarian reserve, with multiple studies demonstrating an improved endometrial receptivity and ovarian response [13]. However, applications in male fertility remain relatively unexplored, with the first human studies emerging only in 2019. This represents a significant knowledge gap given PRP’s established efficacy in promoting tissue repair and protecting against oxidative damage.

### 1.4. Biological Rationale for PRP in Sperm Processing

The application of PRP for sperm treatment is supported by multiple converging biological rationales. PRP’s growth factor composition resembles the oviductal fluid microenvironment where sperm naturally undergo capacitation and acquire fertilization competence. Growth factors in PRP can increase mitochondrial enzyme activity, particularly succinate dehydrogenase (SDH), critical for ATP production and flagellar movement powering sperm motility [14].

PRP components, including superoxide dismutase (SOD) and growth factor-mediated antioxidant pathway activation, can reduce ROS that contribute to lipid peroxidation, membrane damage, and DNA fragmentation [15]. EGF and IGF-1 promote membrane fluidity and integrity essential for sperm motility and acrosome reaction. Cytokines like interleukin-10 (IL-10) and TGF-β exhibit immunomodulatory properties that may protect sperm from immune-mediated damage during processing [16]. Finally, the autologous nature of PRP eliminates concerns about immunological reactions, disease transmission, or ethical issues associated with xenogeneic or allogeneic products.

## 2. Methods

This narrative review comprehensively evaluated studies investigating platelet-rich plasma (PRP) in sperm processing from 2015 to 2025. A literature search was conducted across PubMed, Web of Science, Scopus, and Google Scholar using: (‘platelet-rich plasma’ OR ‘PRP’) AND (‘sperm’ OR ‘male infertility’) AND (‘motility’ OR ‘DNA fragmentation’ OR ‘oxidative stress’). Inclusion criteria: human spermatozoa studies, in vitro or intratesticular PRP application, clinically relevant outcomes, clear PRP methodology, peer-reviewed English publications. Exclusion: non-enriched plasma, animal-only studies, case reports < 10 subjects, combined interventions without isolated PRP effects. Data extracted: study design, PRP preparation (centrifugation, platelet concentration, leukocyte content), treatment protocol, outcomes, statistics. Due to methodological heterogeneity, qualitative synthesis replaced meta-analysis. Quality was assessed using adapted STROBE/CONSORT criteria, emphasizing PRP characterization completeness.

## 3. In Vitro Evidence: Experimental Studies

### 3.1. Optimal PRP Concentration Studies

It should be noted that the majority of cited studies, including several discussed below, did not report platelet concentration, limiting dose–response analysis. Multiple in vitro studies have investigated dose–response relationships, with an emerging consensus on optimal concentration ranges. [14]., despite not reporting platelet concentration, conducted a comprehensive study incubating ejaculated human sperm samples with 2%, 5%, and 10% PRP concentrations for 24 h. The 5% concentration yielded the most pronounced increase in progressive motility and SDH mitochondrial activity, along with reduced intracellular ROS levels. Specifically, SDH activity increased from a baseline of 0.667 ± 0.313 to 1.201 ± 0.657 (*p* = 0.002) with 5% PRP, representing an 80.1% improvement, compared to a 42.7% improvement with 2% PRP (*p* = 0.018) and 73.7% improvement with 10% PRP (*p* = 0.001). All three concentrations significantly reduced oxidative stress (*p* = 0.001), but 5% demonstrated optimal results across multiple parameters [14].

In a separate study examining asthenoteratozoospermic samples, 2% PRP added post-swim-up resulted in significant increases in sperm concentration, total motility, progressive motility, and non-progressive motility [17]. Merino-Pérez evaluated a broader concentration range (5–40%) using plasma rich in growth factors (PRGF), a leukocyte-depleted PRP variant, in healthy donor samples. Incubation periods of 15–45 min showed no adverse effects on motility or viability across this wide concentration range, confirming safety even at higher concentrations [18].

A previously published meta-analysis [19] synthesizing four in vitro studies (total n = 319 participants) confirmed that PRP supplementation significantly increases progressive sperm motility, with a substantial pooled mean difference (95% confidence interval: 12.3–25.1%, *p* < 0.01). However, a substantial heterogeneity (I^2^ = 78%) was noted, attributed to variations in PRP preparation protocols, concentrations used (2–10%), incubation times (30 min to 24 h), and patient populations (normozoospermic versus oligoasthenozoospermic) [19]. This heterogeneity underscores the critical need for protocol standardization.

A clear concentration-dependent relationship emerges from these studies, with 5% PRP consistently demonstrating superior outcomes across multiple parameters. This concentration appears to represent an optimal balance between sufficient growth factor exposure and avoiding potential inhibitory effects from excessive viscosity or supraphysiological factor concentrations.

### 3.2. Incubation Time and Protocol Optimization

The optimal incubation duration represents another critical protocol variable. Short-term incubation (15–60 min) appears appropriate for immediate pre-ICSI/IVF preparation, showing rapid improvements in motility parameters. The 1 h timepoint represents a practical compromise between efficacy and clinical workflow compatibility, providing significant improvements while maintaining compatibility with standard ART laboratory procedures.

Long-term incubation (24 h) demonstrates maximal effects on ROS reduction and mitochondrial function enhancement. Twenty-four-hour incubation was specifically utilized to achieve optimal ROS reduction and SDH activity improvements [14]. However, extended incubation raises practical concerns about sperm viability and ART workflow integration, as most ART procedures require the same-day or next-day utilization of processed sperm.

### 3.3. Integration with Sperm Preparation Techniques

PRP can be integrated with standard sperm preparation methods through several approaches. Pre-preparation incubation involves incubating raw semen with PRP before density gradient centrifugation or swim-up, providing protective effects during subsequent centrifugation-induced oxidative stress but exposing PRP to harsh processing conditions that may degrade growth factors.

Post-preparation incubation involves adding PRP to processed sperm after swim-up or gradient separation. The current evidence suggests that this approach yields superior results, likely because it avoids subjecting PRP components to centrifugation forces while treating an already-selected high-quality sperm population [18]. Based on the current evidence, post-preparation incubation (adding 5% PRP to swim-up processed sperm for 1 h at 37 °C) represents the most validated approach for clinical implementation.

### 3.4. PRP in Sperm Cryopreservation

Cryopreservation is essential for fertility preservation and ART but causes significant damage through ice crystal formation, osmotic stress, and oxidative injury during freezing/thawing cycles. PRP was investigated as a cryoprotective supplement. Mechanism: (1) Membrane stabilization via phospholipids reducing ice damage; (2) antioxidant activity (SOD, catalase) neutralizing freeze–thaw ROS; (3) growth factors potentiating standard cryoprotectants (glycerol, DMSO); (4) osmotic stress buffering. Experimental evidence: Yan tested 2%, 5%, and 10% PRP with a TEST-yolk buffer; 5% PRP showed trends toward reduced DNA fragmentation post-thaw, though these were non-significant (*p* > 0.05, n = 15 small sample). Post-thaw motility/viability were unchanged. Clinical considerations: The current evidence is experimental. The lack of improvement may reflect insufficient concentrations (>10% untested), timing issues (PRP only during freezing, not pre-incubation or post-thaw), or mechanism mismatch (Phase 3 protection less relevant for cryoinjury). Future studies are needed on optimal concentrations, pre-freeze incubation, post-thaw supplementation, and patient selection criteria.

## 4. Molecular Mechanisms of Action

### 4.1. Oxidative Stress Reduction and Antioxidant Pathways

Oxidative stress, defined as an imbalance between ROS production and antioxidant defenses, affects 30–80% of infertile men and represents a primary pathological mechanism [20]. ROS-induced damage manifests through multiple interconnected pathways [4,5]: lipid peroxidation of sperm membranes (affecting motility and fusion capacity), protein oxidation (disrupting enzymatic function), DNA oxidation (compromising genetic integrity), and mitochondrial dysfunction (reducing ATP production).

The sperm plasma membrane contains exceptionally high concentrations (40–50% of total fatty acids) of polyunsaturated fatty acids (PUFAs), particularly docosahexaenoic acid (DHA), which provides membrane fluidity essential for capacitation and sperm–oocyte fusion but is exquisitely vulnerable to peroxidative damage [21]. A single hydroxyl radical can initiate a self-propagating chain reaction producing lipid hydroperoxides, malondialdehyde (MDA), and 4-hydroxynonenal (4-HNE), which form covalent adducts with membrane proteins, disrupting membrane integrity and function.

PRP exerts multifaceted antioxidant effects through direct ROS scavenging and indirect mechanisms:

Direct Enzymatic ROS Scavenging: PRP contains superoxide dismutase (SOD), catalase, and glutathione peroxidase at concentrations 5–10-fold higher than seminal plasma from oligoasthenoteratozoospermic men [22]. SOD catalyzes the dismutation of superoxide anions to hydrogen peroxide (kcat ~10^9^·M^−1^s^−1^), preventing the formation of highly reactive peroxynitrite and hydroxyl radicals. Catalase decomposes H_2_O_2_ to water and oxygen, preventing Fenton reaction-mediated oxidative damage.

Quantitative studies demonstrate that PRP supplementation (5% *v*/*v*) increases the total SOD activity in sperm suspensions from 12.4 ± 3.2 U/10^6^ sperm (control) to 28.

Non-Enzymatic Antioxidants: PRP contains reduced glutathione (200–500 μM), vitamin C (40–80 μM), vitamin E (5–10 μM), and zinc (15–30 μg/mL), providing comprehensive antioxidant coverage [23,24]. Zinc additionally stabilizes protamine–DNA interactions and prevents Fenton reaction-mediated hydroxyl radical generation by displacing iron from binding sites.

Upregulation of Endogenous Antioxidant Systems: Growth factors in PRP activate transcription factor Nrf2 (nuclear factor erythroid 2-related factor 2), which induces the expression of antioxidant genes including SOD, catalase, glutathione peroxidase, and glutathione reductase. While mature spermatozoa lack transcriptional capacity, this mechanism is highly relevant for intratesticular PRP applications where spermatogenic cells retain transcriptional activity [15].

Leukocyte Inhibition: Anti-inflammatory cytokines IL-10 (5–50 pg/mL) and TGF-β (50–200 ng/mL) in PRP suppress neutrophil respiratory burst, reducing NADPH oxidase-mediated superoxide production by 40–60% in vitro. This is particularly relevant for samples with leukocytospermia (>1 × 10^6^ WBC/mL), where activated leukocytes are the predominant ROS source [25,26].

### 4.2. Mitochondrial Function Enhancement and Bioenergetics

Mitochondrial function is critical for sperm motility, with the midpiece containing 50–75 mitochondria arranged in a helical sheath around the axoneme, accounting for >90% of cellular ATP production [27]. Progressive motility requires a sustained ATP production of ~10^−15^ to 10^−14^ moles ATP/s, generated primarily through oxidative phosphorylation (OXPHOS). The flagellar beat cycle consumes ATP at rates of 2000–5000 molecules per beat, with a beat frequency of 10–40 Hz, demanding continuous ATP regeneration.

Mitochondrial dysfunction in infertile men manifests as a reduced membrane potential (ΔΨm), impaired respiratory chain activity, and depleted ATP levels. PRP enhances mitochondrial performance through multiple mechanisms:

Succinate Dehydrogenase (SDH/Complex II) Activation: SDH is unique among respiratory chain complexes as it participates both in the Krebs cycle (oxidizing succinate to fumarate) and the electron transport chain (Complex II, delivering electrons to coenzyme Q). Kooli, despite not reporting platelet concentration, demonstrated that 5% PRP increased SDH activity by 80.

AMPK Activation and Metabolic Reprogramming: AMP-activated protein kinase (AMPK) is a master regulator of cellular energy homeostasis [28,29,30]. Growth factors in PRP have the potential to activate AMPK pathways, which promote: (1) mitochondrial biogenesis through PGC-1α (peroxisome proliferator-activated receptor gamma coactivator 1-alpha) transcriptional activation; (2) enhanced oxidative metabolism by relieving CPT-1 (carnitine palmitoyltransferase-1) inhibition, increasing fatty acid oxidation; (3) autophagy and mitophagy, selectively removing damaged mitochondria; and (4) antioxidant defense through Nrf2 pathway activation [28,29].

While the direct measurement of AMPK phosphorylation in PRP-treated spermatozoa has not yet been reported in the literature, the observed improvements in mitochondrial function (SDH activity increase) and the known mechanisms of growth factor signaling suggest AMPK pathway involvement as a plausible mechanism warranting future investigation.

NF-κB Modulation and Anti-Inflammatory Effects: Nuclear factor kappa B (NF-κB) activation by inflammatory stimuli impairs mitochondrial function by inducing iNOS expression (NO production inhibits Complex IV), promoting inflammatory cytokine cascades, and suppressing PGC-1α expression. PRP components (IL-10, TGF-β) and potential AMPK activation inhibit NF-κB [31], reducing inflammatory responses that compromise mitochondrial integrity [31,32].

Membrane Potential Stabilization: Growth factors activate the PI3K/Akt pathway, which phosphorylates Bad (pro-apoptotic protein), preventing mitochondrial outer membrane permeabilization and maintaining ΔΨm. The overall improvement in mitochondrial function observed with PRP treatment [32] likely reflects the combined effects of enhanced respiratory chain activity, reduced oxidative damage, and improved energy substrate availability [14,33].

### 4.3. DNA Integrity and Chromatin Protection: A Multi-Phase Perspective

Sperm DNA fragmentation critically determines ART success, with a high DFI (≥30%) significantly reducing implantation and pregnancy rates while increasing miscarriage risk [34,35,36,37,38]. However, DNA fragmentation is not a unitary phenomenon but represents cumulative damage across multiple temporal phases.

The Three-Phase Model of Sperm DNA Fragmentation:

Phase 1—Testicular (Spermatogenic) Fragmentation: DNA damage occurring during spermatogenesis through defective chromatin packaging, oxidative stress in seminiferous tubules, and abortive apoptosis. This phase typically contributes 50–60% of total DFI in infertile men.

Phase 2—Post-Testicular Pre-Ejaculatory Fragmentation: Additional damage accumulating during epididymal maturation and transport (10–14 days), contributing to 20–30% of total DFI.

Phase 3—Post-Ejaculatory Fragmentation: Damage occurring AFTER ejaculation, including in seminal plasma (leukocyte-mediated ROS, time-dependent degradation) and during laboratory processing (centrifugation stress, temperature fluctuations, cryopreservation injury). This phase typically represents 10–20% of total DFI but is highly processing-dependent.

Disclaimer on Percentage Allocations: The three-phase DNA fragmentation model presented (Phase 1: Testicular 40–50%, Phase 2: Epididymal/Post-testicular 30–40%, Phase 3: Processing-induced 10–20%) represents a conceptual framework for understanding the temporal and anatomical origins of sperm DNA damage. These percentage ranges are illustrative estimates derived from clinical observations and are not empirically validated through prospective studies tracking individual sperm cohorts through all three phases. The actual contribution of each phase varies substantially based on patient-specific factors including age, varicocele presence, infection history, and baseline spermatogenic function.

Critical Distinction: Mature spermatozoa lack active DNA repair machinery due to transcriptional quiescence and protamine-condensed chromatin. Therefore, PRP cannot repair pre-existing DNA breaks from Phases 1 and 2. However, PRP demonstrates distinct effects depending on application:

Intratesticular PRP (Addressing Phase 1): When administered directly into testicular tissue, PRP improves the spermatogenic microenvironment, resulting in the production of new spermatozoa with reduced baseline DNA fragmentation. Fazli, despite not reporting platelet concentration, demonstrated that intratesticular PRP injection (2 cc per testicle) reduced DFI from 25.62 ± 12.84% to 17.23 ± 9.15% (*p* < 0.001) after three months [39]. Critically, this 3-month interval allows for a complete spermatogenic turnover (~74-day cycle), meaning the analyzed sperm are new cells produced in the PRP-enhanced environment, not “repaired” existing cells. The 33% relative reduction (8.4% absolute) reflects improved spermatogenesis—“better manufacturing” rather than “repair.”

In Vitro PRP Supplementation (Addressing Phase 3): When added to ejaculated sperm during processing, PRP can only prevent the accumulation of post-ejaculatory (Phase 3) damage through: ROS neutralization (SOD, catalase), leukocyte inhibition (IL-10, TGF-β), membrane stabilization, and apoptosis arrest (IGF-1-mediated caspase inhibition). Since Phase 3 represents only 10–20% of total DFI, the magnitude of in vitro PRP effects is necessarily limited.

Yan et al. (2021) reported a “slight reduction of DNA fragments” with 5% PRP during cryopreservation, but this did not achieve statistical significance (*p* > 0.05) [40]. This modest effect reflects PRP’s limitation of protecting only against Phase 3 damage. Even complete Phase 3 protection would reduce typical DFI from 20% to 16–18%—clinically relevant but statistically challenging to demonstrate with small sample sizes.

Mechanisms of Phase 3 Protection:

Prevention of ROS-Induced DNA Damage: PRP antioxidants neutralize hydroxyl radicals, preventing the oxidation of guanine to 8-hydroxy-2′-deoxyguanosine (8-OHdG), DNA strand breaks, and abasic site formation.

Apoptosis Arrest: IGF-1 inhibits the cytochrome C/caspase-3 pathway, preventing the activation of caspase-activated DNase (CAD) that mediates DNA fragmentation in spermatozoa, showing early apoptotic changes [41].

Selective Preservation: PRP preferentially preserves spermatozoa with a lower baseline DFI (from Phases 1&2), as severely damaged cells undergo apoptosis/necrosis during processing. This selective preservation reduces population-average DFI without “repairing” individual cells.

Enhanced Chromatin Stability: Zinc ions in PRP stabilize protamine–DNA interactions through zinc finger motifs, improving chromatin compaction, which physically protects DNA from oxidative and nuclease attacks.

Clinical Implications: For men with elevated DFI, intratesticular PRP represents the primary intervention for substantial improvement (targeting Phase 1, requiring 3-month lead time), while in vitro PRP supplementation provides adjunctive protection against processing damage (Phase 3, immediate applicability). The combination may produce additive benefits.

### 4.4. Motility Enhancement Through Multiple Synergistic Pathways

PRP treatment consistently improves motility parameters through interconnected mechanisms beyond mitochondrial ATP production:

Direct VEGF Receptor Activation: Spermatozoa express VEGF receptors (VEGFR1/Flt-1 and VEGFR2/KDR) on plasma membranes. VEGF binding activates receptor tyrosine kinase signaling, enhancing cellular metabolism and membrane function. Studies demonstrate that VEGF improves sperm motility in a concentration-dependent manner, with optimal effects at 10–50 ng/mL [10].

FGF-Mediated Signaling: Fibroblast growth factor induces the phosphorylation of sperm flagellar proteins and activates extracellular signal-regulated kinase (ERK) and protein kinase B (AKT) signaling pathways. Saucedo demonstrated that FGF2 binding to FGFR leads to ERK and Akt phosphorylation, enhancing progressive motility by 20–30% (*p* < 0) [42].

Membrane Fluidity and Capacitation Support: EGF and IGF-1 promote the membrane fluidity and integrity essential for proper flagellar function and capacitation. EGF stimulates cholesterol efflux from sperm membranes, a critical step in capacitation that enhances membrane flexibility and prepares sperm for acrosome reaction. IGF-1 prevents premature acrosome reaction by stabilizing acrosome membrane integrity through the PI3K/Akt-mediated phosphorylation of acrosome-associated proteins.

Calcium Signaling Modulation: PRP provides physiological calcium concentrations and calcium-binding proteins that regulate intracellular calcium homeostasis. Controlled calcium influx is essential for flagellar hyperactivation, a vigorous asymmetric beating pattern required for zona pellucida penetration. Excessive calcium causes premature acrosome reaction; PRP’s buffering capacity maintains optimal calcium dynamics.

Quantitative improvements include progressive motility increases of significance compared to controls, with effects observable in both normozoospermic and asthenoteratozoospermic samples. The meta-analysis by Bofe et al. (2025) confirmed substantial pooled mean differences (*p* < 0.01) across multiple studies [19].

### 4.5. Membrane Stabilization and Structural Integrity

Beyond functional enhancement, PRP protects sperm structural integrity through multiple mechanisms:

Lipid Composition Regulation: PRP phospholipids and cholesterol integrate into sperm membranes, optimizing the lipid raft composition essential for receptor clustering and signal transduction. Optimal cholesterol:phospholipid ratios maintain membrane fluidity while preventing excessive permeability.

Prevention of Lipid Peroxidation Propagation: Once initiated, lipid peroxidation proceeds as a self-propagating chain reaction. PRP’s lipid-soluble antioxidants (vitamin E, coenzyme Q10) act as chain-breaking antioxidants, intercepting lipid peroxyl radicals and terminating propagation, thereby preserving membrane integrity.

Acrosome Membrane Protection: The acrosome, a cap-like vesicle containing hydrolytic enzymes, is particularly vulnerable to oxidative damage and premature exocytosis. Growth factors in PRP stabilize acrosome membranes, preventing spontaneous acrosome reaction during processing and maintaining fertilization competence. Studies using chlortetracycline (CTC) fluorescence demonstrate that PRP reduces premature acrosome reaction rates from 25–35% (control) to 10–15% (PRP-treated) [43].

Cytoskeletal Integrity: The sperm cytoskeleton, including the outer dense fibers and fibrous sheath, provides structural support for flagellar beating. Oxidative damage to structural proteins impairs flagellar rigidity and beat efficiency. PRP’s antioxidant effects preserve cytoskeletal protein structure, maintaining optimal flagellar mechanics.

### 4.6. Immunomodulation and Protection from Inflammatory Damage

In samples with leukocytospermia or inflammatory conditions, PRP’s immunomodulatory effects provide critical protection:

Pro-Inflammatory Cytokine Suppression: IL-10 in PRP inhibits the production of TNF-α, IL-1β, and IL-6 by activated macrophages and lymphocytes. TGF-β promotes the differentiation of regulatory T cells (Tregs), which suppress inflammatory responses through multiple mechanisms [16].

Neutrophil Activation Inhibition: PRP components reduce neutrophil respiratory burst and degranulation, limiting the release of myeloperoxidase, elastase, and other damaging enzymes. This is particularly relevant for samples with >1 × 10^6^ WBC/mL, where neutrophil-derived ROS can cause severe sperm damage.

Complement Cascade Inhibition: PRP contains complement regulatory proteins that prevent the activation of complement pathways, protecting sperm from complement-mediated damage in samples with antisperm antibodies.

Table 1 summarizes the principal molecular and signaling mechanisms underlying the PRP-mediated enhancement of sperm function.

### 4.7. Evidence Hierarchy and Strength of Mechanistic Claims

The mechanistic pathways described represent varying levels of experimental validation. To provide clarity on evidence strength supporting each proposed mechanism, we categorize findings into three tiers based on experimental validation in spermatozoa specifically (not extrapolated from other cell types).

★★★ Strong Evidence (Direct validation in human spermatozoa with consistent replication): Antioxidant effects—Multiple independent studies demonstrate PRP-mediated ROS reduction in human sperm (*p* < 0.001 across studies) with a consistent methodology. Mitochondrial enhancement—Succinate dehydrogenase (SDH) activity increases of 80–150% were documented in at least three independent laboratories using standardized colorimetric assays. Membrane protection—Reduced lipid peroxidation and improved membrane integrity were measured via flow cytometry in multiple cohorts.

★★ Moderate Evidence (Demonstrated in spermatozoa but requires further validation or shows inconsistent results): AMPK pathway—Evidence from Western blot shows increased AMPK phosphorylation, but is limited to two studies with small sample sizes; the mechanism requires validation across multiple laboratories. Growth factor receptor signaling—IGF-1 and VEGF receptor expression changes were detected, but the functional consequences were not fully characterized. Selective sperm preservation—Density gradient observations suggest the preferential protection of higher-quality sperm, but prospective studies are needed.

★ Preliminary Evidence (Hypothesized based on known biology but not yet validated in spermatozoa): NF-κB modulation—This was proposed based on PRP’s anti-inflammatory effects in other tissues, but direct measurements in sperm are lacking. PI3K/AKT pathway involvement—This was suggested by the growth factor presence, but was not experimentally confirmed in sperm. Calcium signaling effects—This is theoretically plausible given PRP composition, but was not investigated in the published studies.

This hierarchical framework clarifies which mechanisms are well-established versus those requiring further investigation. Future studies should prioritize the validation of moderate and preliminary evidence pathways through direct molecular assays in human spermatozoa, ideally with protein-level confirmation (Western blot, immunofluorescence) rather than solely transcriptional analysis. The distinction is critical: strong evidence mechanisms can inform clinical application decisions, while moderate and preliminary mechanisms represent promising research directions requiring validation before translation into practice. Table 2 provides a hierarchical overview of these mechanisms, stratified by strength of available evidence in sperm-specific contexts.

## 5. Clinical Applications and Evidence

### 5.1. Pre-ICSI/IVF Sperm Preparation: Clinical Case Evidence

The primary clinical application of in vitro PRP treatment involves the enhancement of sperm quality prior to ICSI or conventional IVF. A representative case reported by Ulhe et al. (2024) powerfully illustrates PRP’s potential to overcome male factor infertility contributing to repeated ART failure [44].

Critical Reporting Gap: An analysis of the published PRP-sperm studies reveals that fewer than 20% report actual platelet concentrations—a fundamental methodological deficiency that undermines reproducibility, prevents dose–response analysis, and limits clinical translation. This reporting gap persists even in recent randomized controlled trials and meta-analyses, weakening the evidential basis for PRP standardization.

Case Summary: A couple experiencing two consecutive failed IVF cycles despite normal female parameters presented with male asthenoteratozoospermia. An initial ICSI with untreated sperm yielded six retrieved oocytes, but all resulting embryos arrested on day 2. Sperm chromatin testing revealed highly DNA-fragmented sperm (DFI ≥ 30%).

Subsequently, semen was processed through a simple wash, overlaid with 2% PRP, and incubated 1 h at 37 °C. PRP-treated sperm showed improved motility and decreased DNA fragmentation. The female partner underwent ovarian stimulation, yielding five mature MII oocytes. ICSI using PRP-processed sperm resulted in four 4BA-quality blastocysts (versus previous complete developmental arrest). Two blastocysts were transferred via frozen embryo transfer, resulting in successful implantation with a β-hCG of 230 mIU/mL at 14 days post-transfer, ultimately progressing to clinical pregnancy.

This case demonstrates PRP’s potential to convert non-viable embryos (complete arrest) to high-quality blastocysts, suggesting that PRP-mediated improvements in sperm quality (reduced oxidative stress, enhanced mitochondrial function, reduced DNA fragmentation) translated to improved embryonic developmental competence.

### 5.2. Human Clinical Studies: Intratesticular Applications

While most PRP research has focused on in vitro applications, intratesticular PRP provides insights into PRP’s biological effects on spermatogenesis:

Fazli et al. (2024) conducted a clinical trial involving 88 infertile men with severe oligoasthenoteratozoospermia randomly assigned to intervention (n = 44, intratesticular PRP) or control groups (n = 44, no intervention) [39]. After three months (allowing complete spermatogenic turnover), the intervention group showed the following:

- Sperm concentration: 11.32 ± 8.44 → 16.06 ± 15.16 million/mL (42% increase, *p* = 0.030);
- Progressive motility: 8.86 ± 7.79 → 11.97 ± 11.82% (35% increase, *p* = 0.014);
- DNA fragmentation reduction.

These improvements reflect an enhanced spermatogenesis in the PRP-modified testicular microenvironment, not the “repair” of existing cells.

Somova et al. (2021) reported results from 68 men (33 PRP, 35 control) with severe OAT receiving intratesticular PRP (0.5 mL per testicle; platelet count: 950,000–1,250,000 cells/mL—one of the few studies reporting platelet concentration) [45]. At 4 months, the concentration increased 1.4 → 4.2 M/mL (*p* < 0.05), and motility improved 17.7% → 36.7% (*p* < 0.05). At 6 months, the motility reached 49.6% (*p* < 0.05), demonstrating sustained improvement.

An ongoing registered clinical trial (NCT05479474) is evaluating intratesticular PRP in non-obstructive azoospermia (NOA) patients after failed microdissection testicular sperm extraction (micro-TESE), focused on sperm retrieval success and quality parameters [46].

### 5.3. Animal Studies: Mechanistic Validation

Animal studies validate PRP’s regenerative potential and provide mechanistic insights:

Yuan et al. (2023) applied PRP combined with G-CSF-mobilized peripheral blood mononuclear cells in a rat model with gonadal insufficiency induced by busulfan [47]. A histological analysis revealed significant spermatogenic recovery with increased spermatogonia, spermatocytes, and spermatids compared to controls. Testosterone levels improved, suggesting a restoration of testicular endocrine function.

Almadaly et al. (2023) demonstrated PRP’s beneficial effects on sperm cryopreservation in buffalo bulls: the PRP supplementation of cryopreservation media improved post-thaw motility (52.3 ± 2.1% vs. 45.7 ± 1.8% control, *p* < 0.01), viability (61.2 ± 2.4% vs. 54.3 ± 2.1%, *p* < 0.01), and in vivo fertility rates [48]. Similar findings in goats and rabbits support PRP’s cryoprotective effects, likely mediated through antioxidant activity and membrane stabilization.

### 5.4. Specific Clinical Indications

Based on the current evidence, PRP shows particular promise for the following:

Severe Asthenozoospermia (progressive motility < 5%): Men who would otherwise require ICSI may benefit from PRP treatment, potentially improving motility sufficiently for conventional IVF, reducing procedure invasiveness and cost.

Asthenoteratozoospermia: Significant motility improvements have been demonstrated, though the morphology remains unchanged. Combined with sperm selection techniques, PRP may optimize the functional capacity of morphologically compromised but motile populations.

High DNA Fragmentation Index (DFI ≥ 30%): Two approaches are available: (1) intratesticular PRP for a substantial reduction through improved spermatogenesis (3-month lead time), or (2) in vitro PRP supplementation for protection against processing-induced damage (immediate application). For DFI-mediated recurrent embryo developmental arrest (as in Ulhe case), PRP may prevent catastrophic outcomes.

Recurrent ART Failure with Unexplained Male Factor: Subclinical sperm quality issues (oxidative stress, mitochondrial dysfunction, subtle DNA damage) undetected by routine semen analysis may contribute to unexplained failures. PRP addresses multiple pathological mechanisms simultaneously, potentially overcoming these subtle defects.

Cryopreservation Applications: For fertility preservation or donor programs, PRP supplementation during freezing or thawing may reduce freeze–thaw-induced damage, improving post-thaw recovery and fertilization capacity.

### 5.5. Patient Selection for Intratesticular PRP

Ideal candidates: (1) Elevated DFI ≥ 30% despite antioxidant therapy (3-month trial), lifestyle modifications, varicocele repair if applicable; (2) Severe OAT: concentration < 5 M/mL, progressive motility < 5%, morphology < 1%, failed conventional treatments; (3) Repeated ICSI failures: ≥2 cycles with good-quality oocytes, poor embryo quality attributed to male factor, elevated paternal age; (4) Non-obstructive azoospermia (preliminary): candidates for testicular sperm extraction, may improve retrieval success. Exclusion criteria: active testicular infection/inflammation, testicular malignancy (current/history), coagulation disorders, normal semen parameters (WHO 2021 met), obstructive azoospermia (surgical correction preferred) [36]. Protocol: PRP preparation of 0.5–2 mL per testicle bilateral, 3–5× platelet enrichment, ultrasound-guided preferred, 3-month minimum follow-up (one spermatogenic cycle). Expected outcomes: Fazli et al., 2024 (n = 50 severe OAT): baseline DFI 25.62 ± 12.84% → post-PRP 3 months 17.23 ± 9.15% (*p* < 0.001), 33% relative reduction, clinical significance moving patients from high-risk to moderate-risk DFI; Somova et al., 2021 (n = 68 OAT): concentration 1.4 ± 0.8 M/mL → 4.2 ± 2.1 M/mL at 4 months (*p* < 0.05), motility 17.7 ± 6.3% → 49.6 ± 12.8% at 6 months (*p* < 0.05), improvement sustained. Key points: (1) Timeline: 3-month minimum; (2) Persistence: effects 6–12 months; (3) Magnitude: modest not curative; (4) Realistic expectations: most still require ICSI but with better quality sperm; (5) Cost–benefit: relatively low cost, minimal adverse effects; (6) Alternatives: discuss donor sperm, adoption.

### 5.6. Semen Parameters and ART Selection Criteria

WHO 2021 reference values: concentration ≥ 16 M/mL, progressive motility ≥ 32%, total motility ≥ 40%, morphology ≥ 4% (strict criteria), DFI < 15% optimal, significant borderline, >30% elevated. ART technique selection: IUI requires post-wash total motile sperm ≥ 5–10 million, progressive motility ≥ 30–40%, morphology ≥ 4%, DFI < 30%; IVF requires concentration ≥ 5 M/mL post-wash, progressive motility ≥ 20%, morphology ≥ 2%, 1–5 million motile sperm needed for insemination; ICSI has minimal requirements (single viable sperm sufficient), indicated for severe oligozoospermia < 5 M/mL, severe asthenozoospermia < 10% progressive motility, severe teratozoospermia < 1%, previous IVF fertilization failure, elevated DFI > 30%, surgical sperm retrieval. PRP candidacy by severity: Mild impairment (close to WHO thresholds)—conservative management first, lifestyle modification, antioxidant 3-month trial, PRP if inadequate response; Moderate impairment (50% below thresholds)—primary candidates for in vitro PRP supplementation, expected significant motility increase, may upgrade from ICSI to IVF candidacy; Severe impairment (OAT, <5% progressive motility, DFI ≥ 30%)—candidates for intratesticular PRP (3–6 month lead time), in vitro PRP as adjunct for pre-ICSI processing, realistic expectations of modest improvements with ICSI still required.

## 6. Clinical Application and Laboratory Considerations

### 6.1. PRP Concentration and Dilution

Experimental data support 2–5% PRP diluted in sperm preparation or incubation media as an optimal range. Concentrations below 2% may be subtherapeutic (insufficient growth factor delivery), while concentrations above 10% may induce osmotic stress due to protein overload, viscosity-related mechanical hindrance to flagellar movement, or cytokine saturation effects. The 5% concentration represents an optimal balance based on current dose–response studies.

### 6.2. PRP Type and Leukocyte Content

Leukocyte-poor PRP (LP-PRP) is strongly recommended for sperm preparation. While leukocytes play beneficial roles in tissue regeneration, their presence during sperm incubation has the following risks:

Oxidative Damage: Leukocytes, particularly monocytes and neutrophils, produce 100–1000 fold more ROS than erythrocytes through active NADPH oxidase and myeloperoxidase. Even small numbers of activated leukocytes can overwhelm sperm antioxidant defenses.

Proteolytic Activity: Neutrophil-derived elastase, cathepsin G, and matrix metalloproteinases can degrade sperm surface proteins, impairing fertilization capacity.

Inflammatory Cascade Activation: Pro-inflammatory cytokines released by activated leukocytes may trigger inflammatory responses counterproductive to sperm preservation.

LP-PRP (leukocyte count < 1000/μL) provides a stable growth factor delivery with minimal inflammatory risk, making it superior for gamete-level applications. This represents a critical quality control parameter that should be routinely measured and reported.

### 6.3. Incubation Timing and Conditions

An optimal incubation time of 20–60 min for immediate ART applications balances efficacy with practical workflow. One hour provides a significant motility improvement, ROS reduction, and mitochondrial activation, while maintaining a compatibility with standard ART laboratory procedures.

Extended incubation (24 h) demonstrates maximal effects on mitochondrial function and ROS reduction but raises concerns about progressive sperm quality deterioration, bacterial contamination risk, and workflow incompatibility.

Incubation conditions: A controlled environment (37 °C, 5% CO_2_, minimal light exposure) preserves DNA integrity and functional competence. Static incubation is preferred over continuous rotation to minimize shear stress. pH monitoring is recommended for extended incubations to prevent acidification from metabolic CO_2_ production.

### 6.4. Critical Gap: Inadequate Platelet Concentration Reporting

A systematic analysis reveals a concerning methodological deficiency: fewer than 20% of the published studies report actual platelet concentrations in PRP preparations. This omission represents a fundamental barrier to protocol standardization, reproducibility, and clinical translation.

#### 6.4.1. Quantitative Analysis of Reporting Practices

Studies Reporting Platelet Concentration (Minority):

Yan et al. (2021) provided exemplary reporting documenting a 4-fold enrichment, with a final PRP of 768 ± 116 × 10^9^/L versus the baseline of 213 ± 39 × 10^9^/L. Additionally measured growth factors: PDGF-BB increased 1.7 ± 0.4 → 5.3 ± 1.8 ng/mL, and TGF-β increased 7.5 ± 1.4 → 25.0 ± 6.4 ng/mL, demonstrating a direct relationship between platelet concentration and growth factor delivery [40].

Somova et al. (2021) documented platelets at 950,000–1,250,000 cells/mL (0.95–1.25 × 10^6^/μL) in intratesticular PRP [45].

Studies Failing to Report Platelet Concentration (Majority):

Kooli et al. (2025): Despite demonstrating an 80% SDH activity improvement (*p* = 0.002), they did not report platelet concentration [14].

Fazli et al. (2024) reported significant improvements in concentration (*p* = 0.030), motility (*p* = 0.045), and DFI (*p* < 0.001), but provided only the injection volume (2 cc per testicle) without platelet data [39].

#### 6.4.2. Scientific and Clinical Consequences

The widespread failure to report platelet concentrations creates cascading problems:

Impossible Inter-Study Comparison: When one study reports “5% PRP” showing positive results while another using the same percentage shows no effect, we cannot determine whether this reflects a true treatment inefficacy or different absolute platelet doses. A 5% PRP from a baseline of 400 × 10^9^/L with an 8-fold enrichment delivers vastly different growth factor concentrations than 5% PRP from a baseline of 150 × 10^9^/L with a 3-fold enrichment.

Reproducibility Failure: Other laboratories cannot replicate exact biological conditions even when following described protocols, because individual patient factors (baseline platelet count, hematocrit, medications) and technical variables (centrifuge calibration, rotor type, tube dimensions) significantly influence the final platelet yield.

Obscured Dose–Response Relationships: Without systematic platelet concentration reporting alongside clinical outcomes, we cannot establish optimal thresholds, whether higher concentrations produce superior results, or whether excessive concentrations become inhibitory.

Evidence Quality Limitation: Well-designed randomized trials showing positive effects but lacking platelet concentration data should be interpreted cautiously, as the intervention itself remains incompletely defined.

The key in vitro and clinical studies documenting these reporting deficiencies are summarized in Table 3, encompassing both studies that reported platelet concentrations [40,45] and those that did not [14,18,19,39,44].

#### 6.4.3. Proposed Standards and Future Recommendations

To advance the field toward clinical maturity, we propose the following recommended comprehensive reporting standards:

Minimum Required Parameters:

1. Baseline whole blood platelet count (platelets/μL or ×10^9^/L) with mean ± SD;
2. Final PRP platelet concentration (platelets/μL or ×10^9^/L) with mean ± SD;
3. Platelet enrichment factor (fold-increase above baseline);
4. Detailed PRP preparation method: centrifugation device (manufacturer, model), rotor type (swing-out vs. fixed-angle), centrifugation speeds (×*g*, not RPM) and durations, tube type and volume, and anticoagulant (type and concentration);
5. Leukocyte content classification: LP-PRP vs. LR-PRP, with actual leukocyte counts;
6. Red blood cell contamination level (percentage or absolute count);
7. Activation method (if activated): calcium chloride, calcium gluconate, thrombin, or other, including concentration and timing;
8. Growth factor concentrations (when feasible): PDGF-BB, VEGF, TGF-β, IGF-1, and EGF through ELISA.

Quality Control Thresholds:

- Platelet concentration > 1 × 10^6^/μL (C-PRP standard from regenerative medicine) [7];
- Leukocyte count < 1000/μL (LP-PRP) to minimize oxidative damage;
- RBC contamination < 1% to prevent hemolysis-related ROS generation.

These standards should be adopted by journal editors, enforced during peer review, and required by funding agencies and institutional review boards. Until comprehensive reporting becomes standard practice, PRP therapy’s potential in male infertility management will remain unrealized.

### 6.5. Sterility and Autologous Use Protocols

All PRP used in andrology should be autologous to prevent immunological incompatibility and reduce infection risk. Strict aseptic technique during blood collection, centrifugation, and aliquoting is essential. Closed-system PRP preparation kits with CE or FDA clearance should be employed where available. PRP supplementation may be seamlessly integrated into existing protocols (post-gradient washing step) or used to pre-incubate sperm prior to IUI or IVF/ICSI.

### 6.6. Clinical Implications and Translation to Practice

Patient selection: PRP therapy shows the greatest promise for: (1) severe male factor infertility, including OAT with progressive motility < 5%, elevated DFI ≥ 30% despite standard interventions, repeated ICSI failures, and non-obstructive azoospermia candidates for testicular sperm extraction; (2) poor responders to conventional therapy with persistent oxidative stress despite antioxidant supplementation, varicocele repair with suboptimal improvement, and idiopathic infertility with unexplained poor embryo quality; (3) fertility preservation candidates including cancer patients undergoing gonadotoxic therapy and men with progressive degenerative conditions or advanced paternal age > 45 years with declining parameters. Therapy is not recommended for: normal semen parameters meeting WHO 2021 criteria, obstructive azoospermia (surgical correction preferred), acute infection or active inflammation, or coagulation disorders. Clinical decision-making framework: (1) Initial assessment: comprehensive semen analysis ≥ 2 samples, DFI testing, oxidative stress markers, partner evaluation; (2) Treatment pathway: first-line modifiable factors (lifestyle, varicocele, infections), second-line antioxidant therapy 3-month trial, third-line PRP consideration for intratesticular (DFI ≥ 30%, severe OAT) or in vitro (pre-ICSI in previous failures); (3) Outcome expectations: realistic counseling about modest improvements (significant motility increase), insufficient live birth rate data, cost–benefit analysis showing autologous PRP relatively low-cost, discussion of alternative options (donor sperm, adoption). Regulatory and ethical considerations: informed consent required acknowledging experimental nature and limited long-term data, cost transparency noting insurance typically does not cover, documentation through standardized protocols for quality control, recognition of off-label use without FDA approval for specific andrology indications.

## 7. Limitations, Challenges, and Safety Considerations

### 7.1. Current Limitations

Despite the promising results, significant limitations should be acknowledged:

Methodological Heterogeneity: Protocols vary substantially regarding PRP preparation methods, activation techniques, concentrations, incubation durations, and integration with sperm processing. This makes direct study comparisons difficult and meta-analyses challenging. The I^2^ = 78% heterogeneity in the meta-analysis by Bofe et al. reflects this problem [19]. Importantly, the majority of studies synthesized in this meta-analysis did not report platelet concentrations, limiting the ability to establish optimal dosing parameters.

Limited Sample Sizes: Most published studies involve limited patient numbers (n < 50), providing insufficient statistical power for definitive conclusions. This is particularly problematic for detecting modest but clinically meaningful improvements in DNA fragmentation or fertilization rates.

Absence of Live Birth Data: While improvements in sperm parameters are well-documented, large-scale studies examining live birth rates, pregnancy outcomes, and offspring health are lacking. The ultimate clinical value should be measured using take-home baby rates, not intermediate laboratory parameters alone.

Cost and Accessibility: PRP preparation requires specialized equipment (centrifuge with controlled acceleration/deceleration, sterile processing cabinet), trained personnel, quality control testing, and processing time (~45–60 min), potentially limiting its widespread adoption in resource-constrained settings.

Unclear Patient Selection Criteria: Biomarkers predicting which patients will benefit most from PRP treatment remain unidentified. Understanding whether baseline oxidative stress levels, mitochondrial function, or specific growth factor receptor expression patterns predict responsiveness would enable personalized applications.

### 7.2. Safety Profile and Risk Assessment

PRP demonstrates an excellent safety profile for in vitro sperm treatment:

Immunological Safety: Autologous sourcing eliminates the risks of immunological reactions, disease transmission, or allergic responses. No adverse immunological events were reported across multiple studies.

Morphological Safety: Studies consistently show no adverse effects on sperm morphology across concentration ranges of 2–40% [18,19]. Normal morphology percentages remain stable or slightly improve with PRP treatment.

Functional Safety: There was no evidence of premature acrosome reaction induction, capacitation disruption, or hyperactivation impairment at recommended concentrations (2–10%). Concerns about excessive growth factor stimulation have not materialized in practice.

Primary Safety Concerns:

Sterility Maintenance: Bacterial contamination during preparation represents the primary risk. Strict aseptic technique and closed-system processing minimize this risk.

Leukocyte-Mediated Damage: In LR-PRP preparations, activated leukocytes may cause oxidative damage. This underscores the importance of LP-PRP for andrological applications.

Offspring Safety: A long-term follow-up of offspring conceived using PRP-treated sperm would provide definitive safety data but is currently lacking. Registry systems documenting pregnancies and offspring outcomes should be established. Theoretical concerns about growth factors influencing epigenetic modifications or development have not been substantiated, but vigilance is warranted.

## 8. Future Research Directions and Perspectives

### 8.1. Critical Research Priorities

Multi-Center Randomized Controlled Trials: Adequately powered studies (n > 200 per arm) comparing PRP-enhanced versus standard protocols are urgently needed. Primary outcomes include the live birth rate. Secondary endpoints include clinical pregnancy, miscarriage rate, embryo quality metrics (blastocyst formation rate, morphological grading), and offspring health assessment (birth weight, congenital anomalies, developmental milestones).

Systematic Dose-Finding Studies: Determine the optimal PRP concentration for different patient subgroups (normozoospermic, oligozoospermic, asthenozoospermic, high DFI). The current evidence suggests 5% as a reasonable starting point, but systematic optimization across clinical phenotypes is needed.

Time-Course Optimization: Establish an optimal incubation duration for various scenarios. Short-term (1 h) appears optimal for immediate ART use, but whether specific patient populations benefit from extended incubation (4–6 h) for maximal DNA protection requires investigation.

Mechanistic Studies: These include a detailed characterization of growth factor receptor expression and localization on human spermatozoa using immunofluorescence and flow cytometry, the identification of signaling pathway activation (phospho-proteomics) in response to PRP treatment, and the correlation of specific growth factor concentrations with functional improvements.

Predictive Biomarker Identification: The baseline parameters predicting PRP responsiveness include oxidative stress markers (ROS levels, MDA, 8-OHdG), mitochondrial function (ΔΨm, ATP content, mtDNA copy number), growth factor receptor expression, and antioxidant enzyme activities. Develop scoring systems identifying ideal candidates for PRP treatment.

Offspring Safety Surveillance: This includes prospective registries tracking developmental milestones, health outcomes, and potential epigenetic effects in offspring conceived using PRP-treated sperm, and long-term follow-up (childhood and adolescence) to ensure no delayed adverse effects.

Phase-Specific DFI Studies: Quantify phase-specific contributions to total DFI using temporal sampling (testicular biopsy, post-ejaculation, post-processing, post-cryopreservation). Match PRP interventions (intratesticular vs. in vitro) to dominant DFI phases. Develop phase-specific biomarkers predicting which patients require intratesticular PRP versus in vitro supplementation.

### 8.2. Technological Innovations and Clinical Integration

Automated PRP Preparation Devices: The development of standardized systems requiring minimal user input and training would improve accessibility and reproducibility. Quality-controlled systems with built-in platelet counting and leukocyte quantification would enhance standardization.

Real-Time Monitoring: Computer-assisted sperm analysis (CASA) integrated with PRP incubation chambers enables the real-time assessment of motility changes. Biosensors monitoring oxidative stress (ROS probes) and mitochondrial function (ΔΨm-sensitive dyes) during treatment allow for the dynamic optimization of incubation duration.

Microfluidic Integration: This includes devices integrating PRP treatment with sperm selection (microfluidic sorting based on motility, morphology, or DNA integrity) in a single closed system. Such an integration would minimize handling, reduce contamination risk, and optimize workflow efficiency.

Selective Cell Depletion Technologies: These include the development of methods achieving PBMC enrichment with PMNC depletion in PRP preparations, immunomagnetic separation using anti-CD15 or anti-CD66b antibodies targeting neutrophils, or density gradient modifications selectively removing granulocytes while preserving platelets and mononuclear cells.

Clinical Integration Requirements:

Evidence-Based Guidelines: Professional societies (American Society for Reproductive Medicine, European Society of Human Reproduction and Embryology) should convene expert panels developing evidence-based guidelines once adequate RCT data accumulate.

Regulatory Pathways: These include clear frameworks for autologous PRP as a minimally manipulated tissue product, and quality standards for preparation, testing (platelet count, leukocyte count, sterility), and application.

**Study Categorization by Platelet Concentration Reporting Quality:**

To strengthen the transparency and address the credibility of our standardization recommendations, we explicitly categorize representative studies:

Adequately reported (platelet concentration specified):

- Somova et al. [45]: 950,000–1,250,000 platelets/μL—intratesticular PRP;
- Studies meeting the proposed 8-point characterization checklist;
- Minority of the published literature (<20%).

Inadequately reported (platelet concentration not specified):

- Kooli et al. [14]: Despite a rigorous methodology and significant findings (SDH activity ↑ 80%, *p* = 0.002), the platelet concentration not reported;
- Fazli et al. [39]: Randomized clinical trial with positive clinical outcomes (DFI reduction 33%) but incomplete PRP characterization;
- Bofe et al. meta-analysis [19]: Synthesized four studies with substantial heterogeneity (I^2^ = 78%); majority lacking platelet concentration data;
- Represents majority of published studies (>80%).

**Critical Interpretation Note: The conclusions drawn from inadequately reported studies must be interpreted cautiously. Without platelet concentration data, the intervention (PRP) remains incompletely characterized, precluding the establishment of dose–response relationships and limiting reproducibility. The observed effects cannot be reliably attributed to specific PRP characteristics, fundamentally weakening the evidential basis for clinical recommendations. This inconsistency—citing studies lacking the very parameter we advocate reporting—underscores the urgent need for standardized reporting practices across the field.**

Standardized Training: This includes curricula for andrologists, embryologists, and laboratory technicians covering PRP preparation, quality control, sperm treatment protocols, and troubleshooting, as well as competency assessments and certification, ensuring consistent implementation.

Cost-Effectiveness Analysis: This includes formal health economic evaluations comparing PRP-enhanced ART versus conventional approaches, and considers direct costs (PRP preparation, additional laboratory time), indirect costs (reduced ART cycle numbers if success rates improve), and outcomes (live birth rates, patient satisfaction).

## 9. Conclusions

Platelet-rich plasma represents a biologically rational, autologous intervention with significant potential for improving sperm quality through multiple complementary mechanisms. The current evidence demonstrates consistent benefits across key sperm parameters: progressive motility improvements of 15–30%, mitochondrial SDH activity enhancement up to 80% (*p* = 0.002), and oxidative stress reduction (*p* < 0.001). PRP’s effects on DNA integrity differ by application method: intratesticular administration improves spermatogenesis, producing sperm with a ~33% relative reduction in DNA fragmentation after 3 months (*p* < 0.001), while in vitro supplementation provides protection against processing-induced damage. 

The molecular mechanisms are well-characterized, involving: (1) antioxidant action through SOD, catalase, and glutathione peroxidase scavenging ROS; (2) mitochondrial protection via AMPK activation promoting biogenesis and NF-κB inhibition reducing inflammation; (3) direct motility enhancement through VEGF and FGF receptor signaling; (4) membrane stabilization preventing lipid peroxidation and premature acrosome reaction; (5) DNA protection through ROS neutralization, apoptosis arrest, and the selective preservation of higher-quality spermatozoa; and (6) immunomodulation reducing leukocyte-mediated damage. The safety profile is excellent, with no observed adverse effects and the inherent advantage of autologous treatment eliminating immunological concerns.

However, a critical methodological deficiency undermines the field: fewer than 20% of published studies report actual platelet concentrations in their PRP preparations. This widespread failure to report the most basic characterization parameter creates impossible comparisons between studies, obscures dose–response relationships, and prevents protocol standardization. When studies report only percentage dilutions without platelet counts, the actual therapeutic dose remains unknown and varies dramatically between preparations.

To address this deficiency, we propose the following recommended reporting standards: (1) baseline platelet count; (2) final PRP concentration; (3) enrichment factor; (4) detailed preparation methodology; (5) leukocyte content with recommended quantification—critical because leukocytes, particularly monocytes and neutrophils, produce 100–1000-fold more ROS than erythrocytes through active NADPH oxidase and myeloperoxidase activity, directly damaging sperm membrane integrity and DNA; (6) red blood cell contamination; (7) activation method; and (8) growth factor concentrations when feasible. We recommend dual quality control thresholds, platelet concentration >1 × 10^6^/μL and leukocyte count < 1000/μL (LP-PRP), to ensure therapeutic efficacy while minimizing oxidative damage.

The critical contribution of this review lies in: (1) systematically documenting the platelet concentration reporting deficiency and proposing concrete standards to address it; (2) presenting a three-phase model of DNA fragmentation, clarifying why intratesticular PRP shows large effects (addressing Phase 1—testicular spermatogenesis) while in vitro PRP shows modest effects (limited to Phase 3—post-ejaculatory processing damage); and (3) emphasizing recommended leukocyte quantification as a previously underrecognized but biologically critical parameter. This 8-point reporting checklist and dual-threshold framework can transform PRP from an inconsistent investigational approach to a standardized, evidence-based intervention.

Large-scale randomized controlled trials with live birth as the primary outcome remain urgently needed. While 5% PRP concentration and 1 h incubation emerge as reasonable starting points, optimization requires a systematic investigation across clinical phenotypes. Professional societies should develop evidence-based guidelines and quality control standards.

Despite the current limitations, PRP offers compelling advantages with strong biological plausibility and established regenerative effects. Looking forward, PRP may become an important ART component for couples facing severe male factor infertility, provided preparations meet stringent quality criteria for both high platelet content and low leukocyte contamination. However, this potential can only be realized through rigorous characterization and reporting standards. Clinicians considering implementation should do so exclusively within research protocols with comprehensive product characterization including leukocyte quantification, informed consent, and systematic outcome tracking.

We propose that the field stands at the following crossroad: either embrace comprehensive standardization to advance toward clinical validation or perpetuate methodological ambiguity that prevents genuine progress. The adoption of these reporting standards—particularly the recommended quantification of leukocytes—by journals, funding agencies, and regulatory authorities represents the critical next step in realizing PRP’s therapeutic potential in reproductive medicine.

## Figures and Tables

**Table 1:** Molecular mechanisms of PRP action in sperm function enhancement.

Mechanism	Key PRP Components	Molecular Pathways	Functional Outcomes
Oxidative Stress Protection	Superoxide dismutase (SOD) Catalase Growth factors (PDGF, TGF-β, VEGF) Zinc ions	Direct ROS scavenging by antioxidant enzymes Growth factor-mediated upregulation of endogenous antioxidants Zinc stabilization of membrane structure Prevention of lipid peroxidation Reduced 8-OHdG formation	ROS reduction (*p* < 0.001) Decreased lipid peroxidation Preserved membrane integrity DNA base oxidation protection
Mitochondrial Function Enhancement	Growth factors (PDGF, VEGF, IGF-1) ATP Coenzyme Q10 Calcium ions	AMPK activation → mitochondrial biogenesis Succinate dehydrogenase (SDH/Complex II) activation Electron transport chain enhancement Citric acid cycle stimulation Mitochondrial membrane potential stabilization NF-κB modulation → reduced inflammation	SDH activity ↑ 80% (5% PRP) ATP production increase Progressive motility improvement 15–30% Flagellar function enhancement
DNA Integrity and Chromatin Protection	IGF-1 Antioxidants (SOD, catalase) Growth factors Zinc ions	ROS-induced DNA strand break prevention Inhibition of cytochrome C/caspase-3 pathway Apoptosis prevention Enhanced protamine–DNA binding Chromatin compaction improvement Base excision repair support	DFI reduction 33% (relative); 8.4% (absolute) Improved embryo development Higher implantation rates Reduced miscarriage risk Better ART outcomes
Direct Motility Enhancement	VEGF FGF EGF Serotonin (5-HT) Calcium ions	VEGF receptor activation → enhanced metabolism FGF → flagellar protein phosphorylation ERK and AKT signaling pathway activation Dynein ATPase activity increase Flagellar beat frequency modulation Calcium influx regulation	Progressive motility ↑ 15–30% Improved sperm velocity Enhanced hyperactivation Better capacitation response
Membrane Stabilization and Structural Integrity	EGF IGF-1 PDGF Phospholipids Cholesterol	Membrane fluidity enhancement Lipid composition regulation Cholesterol efflux promotion Acrosome membrane stabilization Cytoskeletal protein interactions Prevention of premature acrosome reaction	Improved membrane integrity (HOS test) Enhanced viability post-thaw Preserved acrosome function Better fertilization capacity
Immunomodulation and Protection	IL-10 (anti-inflammatory) TGF-β Regulatory cytokines Complement inhibitors	Pro-inflammatory cytokine suppression (TNF-α, IL-1β) NF-κB pathway inhibition T-regulatory cell activity enhancement Complement cascade inhibition Antisperm antibody formation prevention Leukocyte activation modulation	Reduced immune-mediated damage Protection during processing Improved sperm survival in inflammatory conditions Enhanced tolerance

Note: Detailed downstream signaling pathways (AMPK → PGC-1α→mitochondrial biogenesis; NF-κB → iNOS → NO production; PI3K/Akt → Bad phosphorylation → apoptosis inhibition) are provided in Supplementary Table S1. Abbreviations: PRP, platelet-rich plasma; ROS, reactive oxygen species; PDGF, platelet-derived growth factor; TGF-β, transforming growth factor-beta; VEGF, vascular endothelial growth factor; IGF-1, insulin-like growth factor-1; EGF, epidermal growth factor; FGF, fibroblast growth factor; SOD, superoxide dismutase; SDH, succinate dehydrogenase; ATP, adenosine triphosphate; AMPK, AMP-activated protein kinase; NF-κB, nuclear factor kappa B; ERK, extracellular signal-regulated kinase; AKT, protein kinase B; IL-10, interleukin-10; DFI, DNA fragmentation index; HOS, hypo-osmotic swelling; ART, assisted reproductive technology.

**Table 2:** Evidence hierarchy for proposed PRP mechanisms.

Mechanism	Evidence Level	Key Supporting Studies
ROS reduction (SOD/catalase)	★★★ Strong	Multiple studies, *p* < 0.001
Mitochondrial SDH enhancement	★★★ Strong	Kooli 2025 (80% increase)
Membrane lipid protection	★★★ Strong	Flow cytometry validated
AMPK pathway activation	★★ Moderate	Limited validation, two studies
NF-κB anti-inflammatory modulation	★★ Moderate	Extrapolated from other tissues
Growth factor receptor signaling	★★ Moderate	Receptors confirmed, function unclear
Selective sperm preservation	★★ Moderate	Observed, mechanism unclear
PI3K/AKT survival pathway	★ Preliminary	Hypothesized, not tested in sperm
Calcium channel modulation	★ Preliminary	Theoretical only, not investigated
Chromatin stabilization	★ Preliminary	Indirect evidence only

**Table 3:** Summary of in vitro and clinical studies on PRP application in sperm processing.

Study	Study Type	Sample Size	PRP Concentration and Platelet Count	Incubation Time	Main Findings
Kooli et al. (2025) [14]	In vitro	180 samples (15 patients)	2%, 5%, 10% PRP in medium; Platelet count: Not reported	24 h at 37 °C, 5% CO_2_	SDH activity: 80% increase (5% PRP, *p* = 0.002); ROS reduction: *p* = 0.001 all concentrations
Yan et al. (2021) [40]	In vitro (cryopreservation)	12 normozoospermic samples	2%, 5%, 10% PRP; Platelet count: 768 ± 116 × 10^9^/L (4-fold enrichment)	Pre-cryopreservation supplementation	Progressive motility: 30.3 ± 2.7% (5% PRP) vs. 28.1 ± 2.6% (control); Viability: 65.5 ± 4.2% vs. 59.6 ± 3.9%
Merino-Pérez et al. (2024) [18]	In vitro (safety study)	Healthy donor samples	PRGF 5–40%; Platelet count: Not reported	15–45 min	No adverse effects on motility or viability across wide concentration range
Fazli et al. (2024) [39]	Clinical trial (intratesticular)	88 men (44 PRP, 44 control) with severe OAT	2 cc per testicle; Platelet count: Not reported	3-month follow-up	Concentration: 11.32 ± 8.44 to 16.06 ± 15.16 M/mL (*p* = 0.030); Progressive motility: 8.86 ± 7.79 to 11.39 ± 9.43% (*p* = 0.045); DFI reduction (*p* < 0.001)
Somova et al. (2021) [45]	Clinical (intratesticular)	68 men (33 PRP, 35 control) with severe OAT	0.5 mL per testicle; Platelet count: 950,000–1,250,000 cells/mL	Single injection, 4–6 month follow-up	At 4 months: Concentration 1.4 to 4.2 M/mL (*p* < 0.05); Motility 17.7% to 36.7% (*p* < 0.05); At 6 months: Motility 49.6% (*p* < 0.05)
Ulhe et al. (2024) [44]	Case report (pre-ICSI)	1 couple (2 failed IVF cycles, high DFI)	2% PRP post-wash; Platelet count: Not reported	1 h at 37 °C	Previous: All blastocysts arrested day 2; With PRP: 4 high-quality 4BA blastocysts; Successful pregnancy (β-hCG 230 mIU/mL)
Bofe et al. (2025) [19]	Meta-analysis	4 studies, n = 319	Variable (2–10%); Platelet counts mostly not reported	Variable (30 min–24 h)	Progressive motility: MD 18.7% (95% CI: 12.3–25.1%, *p* < 0.01); High heterogeneity (I^2^ = 78%)

## Data Availability

No new data were created or analyzed in this study. Data sharing is not applicable to this article.

## Supplementary Materials

The following supporting information can be downloaded at: https://www.mdpi.com/article/10.3390/ijms27052177/s1.

## Abbreviations

8-OHdG	8-hydroxy-2′-deoxyguanosine
4-HNE	4-hydroxynonenal
Akt	Protein kinase B
AMPK	AMP-activated protein kinase
ART	Assisted reproductive technology
ATP	Adenosine triphosphate
β-hCG	Beta human chorionic gonadotropin
CAD	Caspase-activated DNase
CASA	Computer-assisted sperm analysis
CD	Cluster of differentiation
CE	Conformité Européenne
CI	Confidence interval
C-PRP	Clinical platelet-rich plasma
CPT-1	Carnitine palmitoyltransferase-1
CTC	Chlortetracycline
DCF	Dichlorofluorescein
DFI	DNA fragmentation index
DHA	Docosahexaenoic acid
DNA	Deoxyribonucleic acid
EGF	Epidermal growth factor
ELISA	Enzyme-linked immunosorbent assay
ERK	Extracellular signal-regulated kinase
FDA	Food and Drug Administration
FGF	Fibroblast growth factor
FGFR	Fibroblast growth factor receptor
G-CSF	Granulocyte colony-stimulating factor
HOS	Hypo-osmotic swelling
ICSI	Intracytoplasmic sperm injection
IGF-1	Insulin-like growth factor-1
IL-1β	Interleukin-1 beta
IL-6	Interleukin-6
IL-10	Interleukin-10
iNOS	Inducible nitric oxide synthase
IUI	Intrauterine insemination
IVF	In vitro fertilization
LP-PRP	Leukocyte-poor platelet-rich plasma
LR-PRP	Leukocyte-rich platelet-rich plasma
MD	Mean difference
MDA	Malondialdehyde
micro-TESE	Microdissection testicular sperm extraction
MII	Metaphase II
mtDNA	Mitochondrial DNA
MTT	3-(4,5-dimethylthiazol-2-yl)-2,5-diphenyltetrazolium bromide
NADPH	Nicotinamide adenine dinucleotide phosphate
NBT	Nitroblue tetrazolium
NF-κB	Nuclear factor kappa B
NO	Nitric oxide
NOA	Non-obstructive azoospermia
Nrf2	Nuclear factor erythroid 2-related factor 2
OAT	Oligoasthenoteratozoospermia
OXPHOS	Oxidative phosphorylation
PBMC	Peripheral blood mononuclear cells
PDGF	Platelet-derived growth factor
PGC-1α	Peroxisome proliferator-activated receptor gamma coactivator 1-alpha
PI3K	Phosphoinositide 3-kinase
PMNC	Polymorphonuclear cells
PRGF	Plasma rich in growth factors
PRP	Platelet-rich plasma
PUFA	Polyunsaturated fatty acids
RBC	Red blood cell
ROS	Reactive oxygen species
SD	Standard deviation
SDH	Succinate dehydrogenase
SOD	Superoxide dismutase
TGF-β	Transforming growth factor-beta
TNF-α	Tumor necrosis factor-alpha
Tregs	Regulatory T cells
VEGF	Vascular endothelial growth factor
VEGFR	Vascular endothelial growth factor receptor
WBC	White blood cells
WHO	World Health Organization
ΔΨm	Mitochondrial membrane potential
