# Supplementary material for: Platelet-Rich Plasma in Sperm Processing for Assisted Reproductive Technology: Molecular Mechanisms, Clinical Applications, and Future Directions
Source: Int J Mol Sci. 2026 Feb 26;27(5):2177. doi: 10.3390/ijms27052177 (PMC12984228; doi:10.3390/ijms27052177)
Supplement: Supplementary file 1 [file ijms-27-02177-s001.zip › ijms-4096901-supplementary.pdf]

Table S1.
Summary of In Vitro and Clinical Studies on PRP Application in Sperm Processing

Study	Study Type	Sample Size	PRP Concentration & Platelet Count	Incubation Time	Main Findings
Kooli et al. (2025)	In vitro	180 samples (15 patients)	2%, 5%, 10% PRP in medium; Platelet count: Not reported	24 hours at 37°C, 5% CO_2	SDH activity: 80% increase (5% PRP, p=0.002); ROS reduction: p=0.001 all concentrations
Yan et al. (2021)	In vitro (cryopreservation)	12 normozoospermic samples	2%, 5%, 10% PRP; Platelet count: 768±116 × 10^9/L (4-fold enrichment)	Pre-cryopreservation supplementation	Progressive motility: 30.3±2.7% (5% PRP) vs 28.1±2.6% (control); Viability: 65.5±4.2% vs 59.6±3.9%
Merino-Pérez et al. (2024)	In vitro (safety study)	Healthy donor samples	PRGF 5-40%; Platelet count: Not reported	15-45 minutes	No adverse effects on motility or viability across wide concentration range
Fazli et al. (2024)	Clinical trial (intratesticular)	88 men (44 PRP, 44 control) with severe OAT	2 cc per testicle; Platelet count: Not reported	3-month follow-up	Concentration: 11.32±8.44 to 16.06±15.16 M/mL (p=0.030); Progressive motility: 8.86±7.79 to 11.39±9.43% (p=0.045); DFI reduction (p<0.001)
Somova et al. (2021)	Clinical (intratesticular)	68 men (33 PRP, 35 control) with severe OAT	0.5 mL per testicle; Platelet count: 950,000-1,250,000 cells/mL	Single injection, 4-6 month follow-up	At 4 months: Concentration 1.4 to 4.2 M/mL (p<0.05); Motility 17.7% to 36.7% (p<0.05); At 6 months: Motility 49.6% (p<0.05)
Ulhe et al. (2024)	Case report (pre-ICSI)	1 couple (2 failed IVF cycles, high DFI)	2% PRP post-wash; Platelet count: Not reported	1 hour at 37°C	Previous: All blastocysts arrested day 2; With PRP: 4 high-quality 4BA blastocysts; Successful pregnancy (β-hCG 230 mIU/mL)
Liu et al. (2025)	Meta-analysis	4 studies, n=319	Variable (2-10%); Platelet counts mostly not reported	Variable (30 min-24 hrs)	Progressive motility: MD 18.7% (95% CI: 12.3-25.1%, p<0.01); High heterogeneity (I^2=78%)
